# Investigating Wrist-Based Acceleration Summary Measures across Different Sample Rates towards 24-Hour Physical Activity and Sleep Profile Assessment

**DOI:** 10.3390/s22166152

**Published:** 2022-08-17

**Authors:** Athanasios Tsanas

**Affiliations:** 1 Usher Institute, Edinburgh Medical School, University of Edinburgh, NINE Edinburgh BioQuarter, 9 Little France Road, Edinburgh EH16 4UX, UK; atsanas@ed.ac.uk or tsanasthanasis@gmail.com; 2 School of Mathematics, University of Edinburgh, James Clerk Maxwell Building, Peter Guthrie Tait Road, Edinburgh EH9 3FD, UK; 3 Alan Turing Institute, London NW1 2DB, UK

**Keywords:** 24-hour activity profile, actigraphy, Axivity AX3, metabolic equivalents (METs), physical activity, smartwatch, wrist-worn wearable sensor

## Abstract

Wrist-worn wearable sensors have attracted considerable research interest because of their potential in providing continuous, longitudinal, non-invasive measurements, leading to insights into Physical Activity (PA), sleep, and circadian variability. Three key practical considerations for research-grade wearables are as follows: (a) choosing an appropriate sample rate, (b) summarizing raw three-dimensional accelerometry data for further processing (accelerometry summary measures), and (c) accurately estimating PA levels and sleep towards understanding participants’ 24-hour profiles. We used the CAPTURE-24 dataset, where 148 participants concurrently wore a wrist-worn three-dimensional accelerometer and a wearable camera over approximately 24 h to obtain minute-by-minute labels: sleep; and sedentary light, moderate, and vigorous PA. We propose a new acceleration summary measure, the Rate of Change Acceleration Movement (ROCAM), and compare its performance against three established approaches summarizing three-dimensional acceleration data towards replicating the minute-by-minute labels. Moreover, we compare findings where the acceleration data was sampled at 10, 25, 50, and 100 Hz. We demonstrate the competitive advantage of ROCAM towards estimating the five labels (80.2% accuracy) and building 24-hour profiles where the sample rate of 10 Hz is fully sufficient. Collectively, these findings provide insights facilitating the deployment of large-scale longitudinal actigraphy data processing towards 24-hour PA and sleep-profile assessment.

## 1. Introduction

Activities of Daily Living (ADLs) have been intrinsically associated with healthcare outcomes; for example, a range of studies have demonstrated links between day-to-day activities and cardiorespiratory status [1,2,3], sleep with physical and mental health [4,5], and more broadly with disease burden [6]. Assessing ADL including day-to-day Physical Activity (PA) and sleep patterns over the short- to long-term has traditionally been achieved through using self-reports (e.g., using standardized questionnaires and diaries), which are known to be prone to recall and reporting biases [7,8]. Using smartphone apps can potentially be more engaging than paper-based forms, it enables time-stamping participants’ responses, and there is evidence of successful long-term adherence in daily questionnaires even for long periods of time (e.g., over a year) [9]. Nevertheless, there is an increasing understanding and recent work highlighting the need to be moving beyond smartphone apps and integrate additional technologies to facilitate objective, longitudinal monitoring of ADL towards assessing both physical and mental health [10,11].

Advances in sensor design, data acquisition and processing, and smart device connectivities through Bluetooth and other protocols, along with increasing affordability and understanding of the benefits of monitoring one’s physical and mental health, have spurred a boom in the development of wearable health-technology devices [12]. For a succinct description of the evolution of accelerometer-based methods for PA assessment, we refer the reader to Troiano et al. [13]. There is an abundance of consumer wearable devices; wrist-worn wearables (smartwatches) have particularly captivated the attention of the public, making it a multi-billion market which is expected to grow further over the next few years [14]. Wrist-worn accelerometers have shown greater promise in terms of adherence and potential for multiple days’ worth of 24-hour monitoring compared to placing the accelerometers elsewhere, e.g., at the waist or hip [15]. In most cases, the processing of the recorded data relies on proprietary algorithms which are embedded in the devices to provide estimates of steps, floors climbed, calories consumed, and overall sleep quality, aiming to provide overall health indicators. A key challenge with consumer-grade devices is that they lack standardization and often thorough validation [12], which is difficult to establish given that the raw data and algorithms are not made available.

Research-grade wrist-worn wearable devices, on the other hand, provide access to the raw data, thus providing researchers with opportunities to develop advanced algorithms, for example, towards the assessment of PA, sleep, and circadian variability characteristics [4,16]. Early research-grade accelerometers focused on *activity counts*, with different manufacturers using different approaches to record them, and this, in turn, complicated cross-device comparisons [16]. For an outline of additional challenges with the use of activity counts, we refer to Bai et al. [17]. Contemporary research-grade wrist-worn wearables are more advanced: they typically provide measurements of three-dimensional acceleration data and often additional modalities such as ambient light and wrist temperature (see [18,19,20] for examples).

There is intense research interest towards developing algorithms that process raw three-dimensional accelerometry data which are typically provided from contemporary research-grade devices, developing custom-based algorithms. In principle, this means that we can achieve cross-device compatibility and demonstrably compare findings with transparent algorithms that are applicable across different brands of wearables and hence standardize measurements in the field of actigraphy. A key challenge when processing raw three-dimensional accelerometry data is the sheer volume and the need to develop some approach to summarize the data to visualize patterns and enable further processing. To appreciate the size of a dataset, it is useful to consider the typical sample rate, which is 10–100 Hz in actigraphy studies [21]: thus, it can be easily inferred that we can collect 1 TeraByte (TB) of data for a single participant within a few weeks. Then it becomes obvious that researchers may be confronted with many TeraBytes of data for a moderate number of participants when collecting longitudinal actigraphy data.

The first step before analyzing the three-dimensional accelerometry data is typically the application of an algorithmic approach to project the recorded acceleration data onto a single vector, which is easier to process. For convenience, we refer to these algorithmic approaches as *acceleration summary measures* in this study. Other studies have used the terms ‘metric’ or ‘activity metric’ instead; however, arguably, this might create some confusion when considering the strict definition in mathematics of a metric, and, therefore, we avoid it here. Intuitively, the acceleration summary measures need to meaningfully summarize the three-dimensional acceleration data in *epochs*, which, in actigraphy studies, are usually per-minute assessments [3,18,21]. There is an ongoing debate on the merits of different acceleration summary measures both in terms of their robustness (for example, considering sensor properties and potentially adjusting for temperature changes) and also in terms of their subsequent use to facilitate further accurate inferences of PA-related outcomes [20,21,22].

In practice, we often want to process the three-dimensional acceleration data to provide insights into PA. One of the most frequent measures assessing the extent of PA in lab-based settings is via the computation of Metabolic Equivalent Tasks (METs), which provide a meaningful and practical procedure for expressing the energy cost of physical activities as a multiple of the resting metabolic rate [1]. Often we use discretized bands of METs to define activity behaviors, where the categorization into (i) sleep, (ii) sedentary PA, (iii) light PA, (iv) moderate PA, and vigorous PA are often used and have been integrated into PA guidelines to promote well-being, including by the World Health Organization (WHO) (https://www.who.int/initiatives/behealthy/physical-activity, last accessed on 30 June 2022) and other health organizations related to morbidities and mortality [23]. Given how important and practical this five-level PA categorization is and the inherent link of acceleration with PA, many researchers have proposed algorithmic methods to infer these (or a subset of these) five levels, using the three-dimensional acceleration data. Traditionally, this has been achieved by using acceleration summary measure thresholds [8,24,25] or building statistical machine learning models [3]. Whereas there is a valid argument to be made for using advanced statistical machine learning models if that approach improves PA categorization estimates substantially over threshold-based methods [3,26], there is still value in pursuing threshold-based approaches because they are arguably more generalizable and easier to interpret and also can serve as useful performance benchmarks for more advanced methods [27].

A further topic of crucial importance which has not been sufficiently carefully studied in the context of actigraphy studies is the use of different sample rates. Indicatively, a large systematic review study aiming to provide practical considerations for actigraphy studies endorsed using the highest sample rate possible, e.g., 90 or 100 Hz [15]. This recommendation is questionable from a practical perspective because there is an inherent trade-off between the sample rate and data-capture duration. Furthermore, from a general signal-processing perspective and following Nyquist’s theorem [28], we know that we need to sample the data with at least twice the maximum frequency of interest for the task at hand. Resorting to the default highest sample rate that contemporary research-grade accelerometers provide (typically 100 Hz) may be excessive for practical applications for the purpose of assessing PA and sleep, and it, indeed, drains the memory of the device faster, thus prohibiting the collection of longitudinal data in a single device charge. This can be a considerable practical limitation if we want to design longitudinal actigraphy studies, where it would be advisable to strive to use the lowest sample rate that can provide sufficiently good data for the purposes of a particular study. Surprisingly, hitherto there have been few studies systematically investigating the effect of sample rate upon the further analysis of raw three-dimensional accelerometry data for wrist-worn devices, e.g., for different accelerometer summary measures and for specific tasks such as PA and sleep assessment. Most of the studies investigating the effect of sample rates have focused on activity counts, e.g., [29]. Clevenger et al. [30] investigated the effect of two sample rates (30 and 100 Hz) on acceleration and activity counts on both hip- and wrist-worn accelerometers. They found that there were differences between the 100 and 30 Hz activity count data; however, they reported that there were no considerable differences in the acceleration data for the wrist-worn accelerometers and that there was excellent agreement (99%) in the estimated PA categories between the two sample rates. It is noteworthy that their study focused on children between the ages 7.3 and 12.5 years, so it does not automatically transpire that these findings necessarily generalize for adults. A further open question is whether using a lower sample rate (e.g., the lowest that many research-grade accelerometers provide, 10 Hz) would still be sufficiently good for sleep and PA assessment, whilst ensuring that we can maximize the data-capture duration in a single accelerometer charge of a wrist-worn device.

Therefore, the motivation for this study was two-fold: (1) explore acceleration summary measures towards estimating PA and sleep, using interpretable threshold-based approaches; and (2) explore whether we need a very high sample rate in the raw three-dimensional acceleration data to accurately estimate sleep and the PA categories. The second part is guided by our ongoing work aiming to collect many weeks’ worth of data in a single device charge to infer sleep and PA towards monitoring healthcare outcomes, and hence we wanted to explore whether the minimum typical sample rate (10 Hz) is sufficient. The aims of the study are, thus, as follows: (a) introducing a new acceleration summary measure, which is usefully summarizing the raw three-dimensional data; (b) empirically investigating the performance of the new acceleration summary measure, along with three well-established acceleration summary measures towards PA assessment; and (c) comparing performance in PA assessment when using raw accelerometry data sampled at four different sample rates, namely 10, 25, 50 and 100 Hz, to explore the generalizability of the proposed algorithms and establish whether we need the high-resolution data for sleep and PA assessment.

## 2. Materials and Methods

### 2.1. Data

This study used a publicly available resource which was released recently, the Comparing Annotated Pictures with Time-Use Diaries’ Recording of Events over 24-hours (CAPTURE-24) dataset [31]. CAPTURE-24 received ethics approval from the University of Oxford Inter-Divisional Research Ethics Committee (IDREC), reference number SSD/CUREC1A/13-262. CAPTURE-24 contains data from 151 participants (52 males), aged between 18 and 91; in the public version of the database, the ages are available in discretized age categories to protect participant confidentiality, and, hence, we cannot provide more detailed summary demographics beyond those already reported in the original study. 

Participants wore the Axivity AX3 wrist-worn activity tracker (https://axivity.com/product/ax3, last accessed on 30 June 2022) on their dominant hand for approximately 24-hours, recording three-dimensional acceleration. Concurrently, they also wore Vicon autograph wearable cameras to record images automatically at 20-to-30-second intervals: in total, approximately 1500–2000 images were typically recorded for each participant during the hours they were awake. Participants also kept a time-use diary and recorded their sleep by using Whitehall sleep diaries. The images and the diaries were reviewed by participants and the CAPTURE-24 researchers to obtain the ground truth activities both regarding sleep and PA behaviors. Moreover, the developers of the database also attached appropriate estimated METs to each diary/camera event [31], building on the widely accepted and used compendium of physical activities providing METs estimates across diverse tasks [32]. Thus, CAPTURE-24 provides concurrent three-dimensional acceleration, along with detailed labels in the form of METs and PA behaviors. For three participants, there are no available labels in CAPTURE-24; hence, they were excluded from further analysis. The labels are provided on a per-minute basis, and in total, we processed 159,008 labeled actigraphy minutes across the 148 participants.

The Axivity AX3 has a configurable sample rate, providing time-stamped entries measuring acceleration across three axes in terms of gravitational force (g). In CAPTURE-24, three-dimensional accelerometer data were recorded at 100 Hz, with a dynamic range of ±8 g (‘g’ is a gravity unit, 1 g = 9.81 m/s^2^). We remark that, although Axivity AX3 can also record ambient light and wrist temperature, these modalities are not available in the public release of CAPTURE-24.

The CAPTURE-24 dataset is publicly freely available from https://ora.ox.ac.uk/objects/uuid:99d7c092-d865-4a19-b096-cc16440cd001 (https://doi.org/10.5287/bodleian:NGx0JOMP5, last accessed on 30 June 2022). For further details and background on the CAPTURE-24 study, we refer readers to Gershuny et al. [31] and Willets et al. [33].

### 2.2. Defining Ground Truth Labels

The developers of CAPTURE-24 used the diaries and METs thresholds to define four activity behaviors in their recent study [3]: (1) *sleep*, indicating the time someone is asleep; (2) *sedentary PA*, indicating the person is awake in a sitting, lying, or reclining posture with energy expenditure lower than 1.5 METs; (3) *light PA*, indicating the person is awake, with energy expenditure being at least 1.5 METs and lower than 3 METs; and (4) *moderate-to-vigorous PA* (MVPA), with energy expenditure 3 METs and above. In this study, we also wanted to distinguish between moderate PA and vigorous PA—this is a meaningful differentiation in large community studies assessing healthcare outcomes [32], including in PA guidelines published by WHO. Vigorous PA is characterized by a large increase in breathing and increased heart rate (e.g., indicating some vigorous sport activity) [32]. Practically, in CAPTURE-24, this was achieved by identifying entries which were previously labeled as MVPA and re-labeling those entries that corresponded to METs 6 and above to indicate vigorous PA, in accordance with the standard approaches used in the literature [32,34]. Thus, in this study, we have five activity categories (sleep; sedentary, light, moderate, and vigorous PA) which are used as the ground truth. For convenience, henceforth we collectively refer to these five as the *PA categories*, comprising sleep and the remaining four *PA levels*. In all cases, the ground truth was summarized in 1-minute epochs to match the corresponding acceleration summaries that are described next (see Section 2.3) and to be consistent with the assessment resolution reported previously using the CAPTURE-24 dataset [3] and similar studies in actigraphy and PA assessment [22].

### 2.3. Actigraphy Data Processing

**Figure 1:**
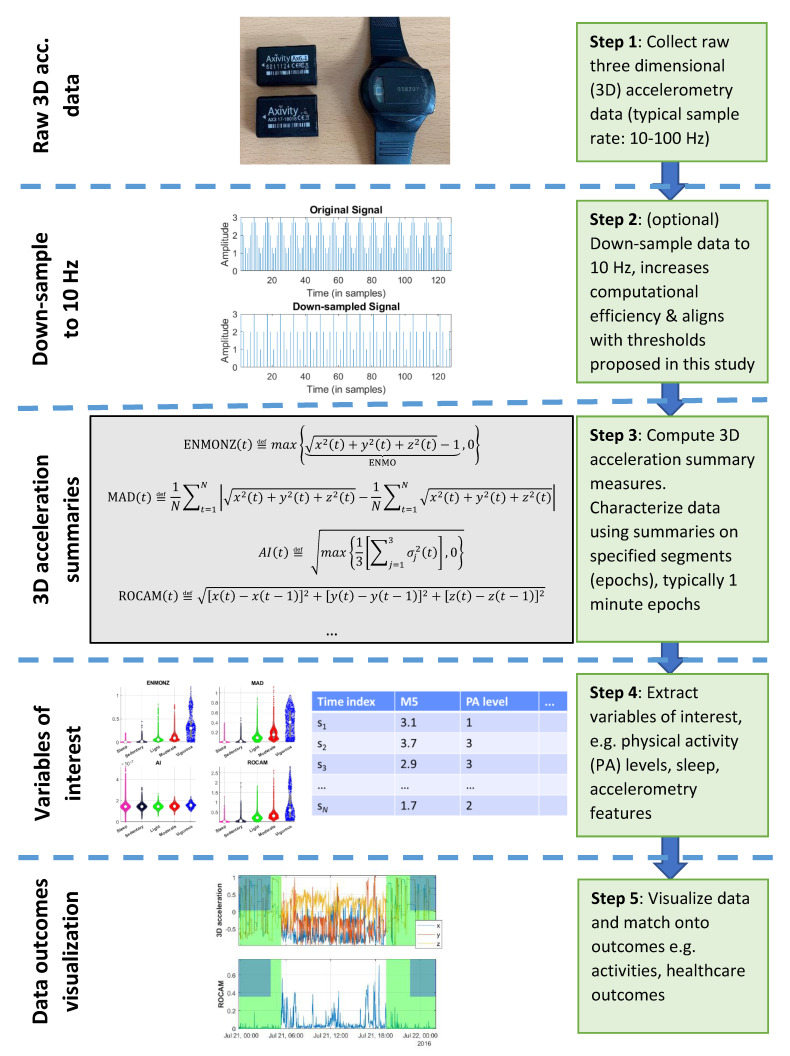
Flowchart with an overview of the methodology used in the study (Steps 2–4) and how it could be deployed in more generic applications, e.g., with healthcare outcomes (Step 5), including sleep-related outputs (see [18]).

The methodology used to process the actigraphy data is graphically illustrated in the flowchart of Figure 1. In the following sections, we describe in detail the following methodological steps: finding a computationally efficient way to summarize the accelerometry data for further processing, using the processed accelerometry data to map onto activities of interest (here, the PA categories), and assessing how well the findings might generalize in new unseen data.

#### 2.3.1. Data Preprocessing

As mentioned above, the sample rate for the three-dimensional acceleration data in CAPTURE-24 is 100 Hz. This leads to a very large file that we want to process in order to match it with outcomes of interest at a chosen resolution (*epoch-length*) and characterize sleep (and potentially sleep patterns), and, if accelerometry data are provided over multiple days, we could also extract diurnal characteristics (see [18]). In principle, we could proceed with characterizing the three-dimensional acceleration by using the methods described in the following sections, and, indeed, the previous studies reporting on CAPTURE-24 have retained the original sample rate [3,33].

Here, we wanted to investigate both processing the raw three-dimensional accelerometry data sampled at 100 Hz and down-sampled versions of the data prior to any processing (Step 2 in Figure 1) for two reasons: (i) computational efficiency, and, more important, (ii) to enable the generalization of the current study’s findings across studies that collect three-dimensional acceleration at lower sample rates. It is important to emphasize that thresholds and models developed by using three-dimensional acceleration data which were sampled at a specific sample rate are not directly generalizable when using different sample rates. In practice, studies collecting raw three-dimensional data configure the sample rate to be typically in the range of 10–100 Hz. There is an inherent trade-off of sample rate and data-collection duration (typically, a smaller sample rate enables longer data collection). For example, in our ongoing actigraphy studies (which will be reported in our future follow-up work), we use a sample rate of 10 Hz because we want to collect longitudinal data; similarly, some other studies use intermediate sample rates (e.g., 25 Hz), depending on their specific needs. Therefore, we have explored four indicative sample rates here: 100, 50, 25 and 10 Hz. We wanted to investigate whether there are objective differences to justify the use of higher-resolution data for the purpose of assessing the PA categories and, in particular, focus on the investigation when data have been sampled at 10 Hz: the implication is that studies which use higher sample rates could always down-sample data to 10 Hz as a standardized sample rate for their analyses. We used a standard finite impulse response filter with anti-aliasing for the down-sampling of the original 100 Hz in CAPTURE-24 to resample the original accelerometry data to different sample rates (50, 25 and 10 Hz) before any further processing and independently repeated the steps that are described in the following sections.

We proceeded with the further processing of the data independently each time (Steps 3 and 4 in Figure 1), as if having four different datasets with the three-dimensional accelerometry data sampled at each of those four sample rates.

#### 2.3.2. Summarizing Three-Dimensional Actigraphy Data

Most contemporary acceleration-based commercial devices which provide access to the raw data record three-dimensional raw acceleration signals, such as the Axivity sensors (used in this study), the Geneactiv sensors (used in similar actigraphy-based healthcare studies, e.g., [18]), and the ActiGraph sensor, which has also been used in similar PA-related studies using actigraphy, e.g., [22]. For convenience, we use the conventional notation x,y,z to denote each of the three axes. In some applications, it might be preferable to focus on one of the three original acceleration axes for a specific purpose; however, in most applications, we want to find an expedient approach to summarize activity across all three axes.

The Euclidean Norm (EN) of the three-dimensional accelerometer (vector magnitude) is arguably the most intuitive and straightforward approach to project the information from the three vectors (3D acceleration) into a single vector and is defined as the square root of the sum of the squared values along the three axes. Van Hees et al. [20] suggested removing the earth gravitational acceleration (1g) from EN, thus developing the Euclidean Norm Minus One (ENMO). Moreover, to inhibit negative subtraction results in ENMO, most studies further enforce non-zero ENMO outputs, resulting in the ENMONZ acceleration summary measure. ENMONZ is probably the most widely used acceleration summary measure currently and is the default in the GGIR software package (https://cran.r-project.org/web/packages/GGIR/index.html, last accessed on 30 June 2022), which has been widely used in research actigraphy studies. The previous studies reporting on CAPTURE-24 have similarly used ENMONZ [31,33]. Algorithmically, ENMONZ is computed by using Equation (1).

(1) $$\mathrm{ENMONZ}\left(t\right)\overset{\mathrm{def}}{=}max\left\{\underset{\underset{\mathrm{ENMO}}{⏟}}{\sqrt{x^{2}\left(t\right)+y^{2}\left(t\right)+z^{2}\left(t\right)}-1},0\right\}$$

where t refers to the time index, and the operator $max\left\{q,0\right\}$ indicates that we use the maximum value between a scalar quantity q and 0, i.e., always obtain non-negative values. Parenthetically, many studies use the algorithmic definition in Equation (1), and, for simplicity, they still refer to it as ENMO, e.g., [22,33].

The mean amplitude deviation (MAD), also known as Vector Magnitude Count (VMC), was proposed by Vähä-Ypyä et al. [35]. It can be thought of as an extension of ENMO in the sense that it uses the mean EN over a number of samples N to reduce the effect of the constant gravitational acceleration.

(2) $$\mathrm{MAD}\left(t\right)\overset{\mathrm{def}}{=}\frac{1}{N}\sum_{t=1}^{N}\left|\sqrt{x^{2}\left(t\right)+y^{2}\left(t\right)+z^{2}\left(t\right)}-\frac{1}{N}\sum_{t=1}^{N}\sqrt{x^{2}\left(t\right)+y^{2}\left(t\right)+z^{2}\left(t\right)}\right|$$

The Activity Index (AI) was proposed by Bai et al. [21] and is a measure of the relative amplitude of activity across the three axes compared to rest. We followed the recommendation of Bai et al. [21] and the follow-up work by the same group [22] wherein AI was originally computed on 1-second epochs, and, subsequently, these entries were summed to obtain the final 1-minute epochs.

(3) $$\mathrm{AI}\left(t\right)\overset{\mathrm{def}}{=}\sqrt{max\left\{\frac{1}{3}\left[\sum_{j=1}^{3}\sigma_{j}^{2}\left(t\right)\right],0\right\}},\;\mathrm{where}\;\sigma_{j}^{2}\left(t\right)=\frac{1}{N}\sum_{t=1}^{N}{\left[a_{j}\left(t\right)-\frac{1}{N}\sum_{t=1}^{N}a_{j}\left(t\right)\right]}^{2}\;\mathrm{where}\;j=x,y,z\;(i.e.,\;\mathrm{one}\;\mathrm{of}\;\mathrm{the}\;\mathrm{three}\;\mathrm{axes})\;\mathrm{and},\;\mathrm{hence},\;\mathrm{computes}\;\mathrm{the}\;\mathrm{corresponding}\;\mathrm{equation}:\;\sigma_{x}^{2}\left(t\right),\sigma_{y}^{2}\left(t\right),\sigma_{z}^{2}\left(t\right).$$

The fourth acceleration summary measure that we propose in this study is referred to as Rate of Change Acceleration Movement (ROCAM) and is an extension of what was recently proposed in our previous work [18,36]. In those studies, we reported that, empirically, this acceleration summary measure led to visually appealing results and had a sound conceptual basis, without any further empirical justification or comparison against competing approaches.

(4) $$\mathrm{ROCAM}\left(t\right)\overset{\mathrm{def}}{=}\sqrt{{\left[x\left(t\right)-x\left(t-1\right)\right]}^{2}+{\left[y\left(t\right)-y\left(t-1\right)\right]}^{2}+{\left[z\left(t\right)-z\left(t-1\right)\right]}^{2}}$$

ROCAM could be conceptually likened to signal processing algorithms that capture the instantaneous energy of a signal (for example, see [37,38]); hence, we empirically found that a postprocessing median filtering would smoothen abrupt instantaneous acceleration variability. For that reason, the output of Equation (4) is then passed through a median filter of length that is equal to the number of samples comprising a 1-second window. Henceforth, we refer to ROCAM as the output of Equation (4), followed by this median filtering. Conceptually, ROCAM is similar to the standard Euclidean distance using the three-dimensional data, with the additional twist that successive differences of the raw acceleration entries are used for the three axes. Using the rate of change focuses on the short-term local changes along the three axes and overcomes the need for prior calibration and removing gravitational effects, as, for example, in ENMONZ and other accelerometry summary measure variants explored by van Hees et al. [20].

In all cases, the three-dimensional acceleration data were summarized on 1-minute epochs by taking the average of the intermediate values computed for each acceleration summary measure and are in gravitational units (g). The following steps are about using the 1-minute epochs of the accelerometry data to match onto the (healthcare) outcomes of interest, which, in this study, are the PA categories.

#### 2.3.3. Estimating Non-Wear Time from Actigraphy

Before we proceed with estimating the PA categories (sleep and PA levels), it is important to exclude segments of non-wear time. When the wrist-temperature is available (increasingly frequently available in many actigraphy studies), this considerably facilitates the detection of non-wear periods. Here, we used the same approach as the developers of CAPTURE-24, where stationary episodes lasting for at least 60 min were identified as device non-wear [31].

#### 2.3.4. Estimating Sleep from Actigraphy

We used the sleep detection algorithm we had previously introduced which we had optimized to detect sleep onset, offset, and awakenings in both controls and people who had been diagnosed with posttraumatic stress disorder, in which sleep disturbances are a hallmark of the condition [18]. In brief, the sleep detection algorithm we had previously proposed works in two steps: first detecting good sleep candidates by using ROCAM and an additional sleep-specific acceleration summary measure we had previously defined for short signal segments [18], and subsequently a postprocessing step to join signal segments detected as sleep candidates. The key difference compared to our original algorithm [18] is that we removed the requirement of the rolling 5-minute average light being below a threshold, since the light modality is not available in CAPTURE-24.

#### 2.3.5. Estimating PA Categorization from Actigraphy

Subsequently, the segments which are not marked as ‘non-wear’ or ‘sleep’ need to be matched onto the four remaining PA categories (sedentary, light, moderate, and vigorous PA). We aimed to achieve this by determining optimized thresholds on a minute-by-minute basis for the acceleration summary measures, on the basis that this provides a generalizable and easily interpretable method, thus indirectly serving to assess the validity of the different acceleration summary measures across the different sample rates.

As a first step, the PA categories can be estimated by defining appropriate thresholds of the acceleration summary measures focusing directly on the minute-by-minute acceleration summary entries with ENMONZ, MAD, AI, and ROCAM. These thresholds were determined by using constrained nonlinear optimization [39], where the optimizer aimed to maximize the overall accuracy (see next section) for the PA differentiation. We clarify that the optimization algorithm did not aim to also differentiate sleep, which was separately estimated (see Section 2.3.4, ‘Estimating Sleep from Actigraphy’). The initializations were determined by following the computations of the densities via diffusion [40] and algorithmically determining thresholds that maximally separate densities, so these are very reasonable first tries to explore optimizing thresholds. The optimization boundaries to guide the optimization algorithm were provided following inspection of the stratified probability distributions of the acceleration summary measures for each PA category.

Finally, we wanted to explore whether there is any additional value in combining the outputs of the threshold-based methods for the different acceleration summary measures. To assess this, we trained a Random Forest (RF) [41,42] to combine the estimates of the PA categories coming out from the application of the threshold methodology for the four acceleration summary measures in order to assess whether their combination further improves accuracy. The RF operates by aggregating the outputs of multiple *base learners* (decision trees) which are trained in parallel by using perturbed versions of the training data obtained via bootstrapping. We used the default options in the RF for the choice of its hyper-parameters: using 500 decision trees and selecting the best feature at each branch from the randomly selected subset of available features (computed by the square root of the original data dimensionality). For further details on RF, we refer the reader to Breiman [41] and Hastie et al. [42]. The reason we chose RF is that this is a robust statistical learning algorithm that can learn complicated nonlinear relationships in the data and which has been described as the ‘best off the shelf’ algorithm because it is very robust to the choice of its hyper-parameters, in stark contrast with other popular statistical learners [42]. We used three approaches to investigate whether the use of RF might improve on the use of thresholds. Firstly, we used the optimized thresholds for each of the four acceleration summary measures independently, and then we presented their outputs (estimated PA categories) as inputs to train an RF. Secondly, we presented directly the four acceleration summary measures as input to train an RF. Thirdly, we jointly presented the estimated PA categories (using the optimized thresholds) for the four acceleration summary measures and the raw acceleration summary measures as input into an RF.

The MATLAB source code for all methods is freely available at the author’s Github page that is dedicated to this project: https://github.com/ThanasisTsanas/ActigraphyToolbox (last accessed on 30 June 2022).

### 2.4. Statistical Analysis

We used the Spearman correlation coefficient to compute the statistical association strength between the four acceleration summary measures (ENMONZ, MAD, AI, and ROCAM) and the PA categories. We denote a statistical relationship between two variables as *statistically strong* when the magnitude of the correlation coefficient is 0.3 and above, following standard recommendations in healthcare applications [43]. Similar methodologies employing the Spearman correlation coefficient to assess the statistical association of the accelerometry-based data and the PA levels were used in related studies previously [8].

We used the two sample Kolmogorov–Smirnov (KS) statistical hypothesis test to assess whether there are statistically significant differences when comparing distributions, assessing statistical significance at the *p* = 0.01 threshold. The null hypothesis is that the two distributions are equal, so if the computed probability, *p*, is below the chosen threshold, we then reject the null hypothesis and accept the alternative hypothesis suggesting that the two distributions are statistically significantly different.

### 2.5. Model Assessment and Generalization

As already indicated above, the sleep detection algorithm was described in our previous work; therefore, we can directly compare both the minute-by-minute ground truth segments marked as sleep against the estimates from the algorithm and also directly compare the sleep onset and sleep offset by following the approach we previously used [18]. For the PA levels, given that we obtained the thresholds from the CAPTURE-24 data, we needed to develop an appropriate strategy. Specifically, we evaluated the performance by using leave-one-participant-out validation, following a similar assessment methodology to Walmsley et al. [3]: the thresholds were optimized by using data from *N*-1 participants, and the overall model performance is averaged from the successive participant left out. 

We used confusion matrices to report the overall performance in terms of estimating the PA categories. For convenience, the information in the confusion matrix is summarized by using accuracy to assess the overall agreement of estimates and ground truth, following the same approach as in the original study reporting on the use of CAPTURE-24 and assessing activity categories [3]. The confusion matrices and accuracies were computed per participant (out of sample data) and averaged across the *N*th repetitions.

## 3. Results

Table 1 summarizes the Spearman correlation coefficients of the four acceleration summary measures explored here (ENMONZ, MAD, AI, and ROCAM) with the five PA categories (sleep; sedentary, light, moderate, and vigorous PA). For convenience, we report these correlations for four different versions of the CAPTURE-24 dataset where we have used the raw 100 Hz data and the down-sampled versions (50, 25 and 10 Hz) of the three-dimensional accelerometry data.

We remark that all four acceleration summary measures exhibit statistically strong associations with PA categories (Spearman correlation coefficients well above |0.3|). Therefore, this is a reassuring finding, as it shows that all four measures capture useful information in the raw three-dimensional acceleration data towards estimating the PA categories used herein. ENMONZ is the least correlated acceleration summary measure and ROCAM is consistently the most strongly associated acceleration summary measure with the PA categories.

A further interesting finding in Table 1 is that the correlations of each of the acceleration summary measures with the PA categories are not drastically affected as a result of using lower-resolution data. ENMONZ exhibits a slightly lower correlation when the accelerometry data are sampled at a low sample rate (10 Hz); however, the other established acceleration summary measures (MAD and AI) appear to be largely unaffected. On the contrary, ROCAM appears to improve somewhat with lower sample rate; this is, perhaps, a counter-intuitive finding, and we revisit it later, in the Discussion.

**Figure 2:**
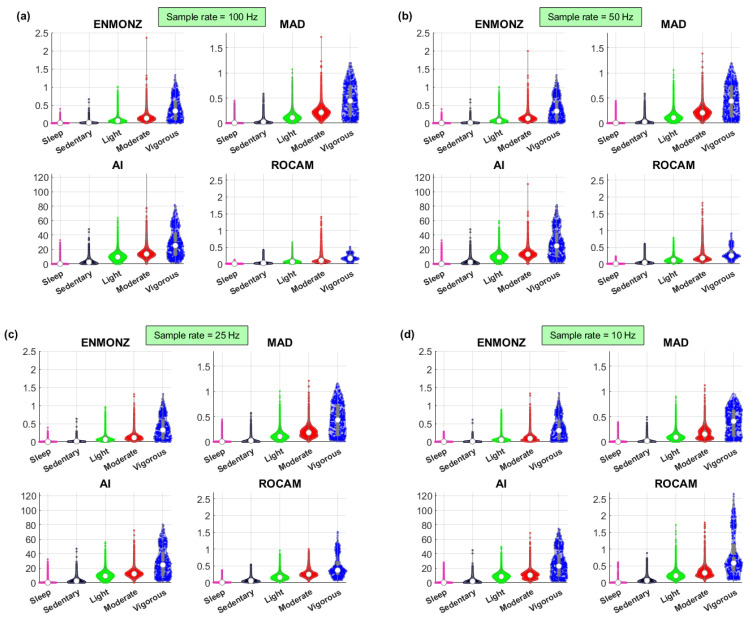
Violin plots to visually illustrate the differences in the acceleration summary measures with the CAPTURE-24 data sampled at (**a**) 100 Hz (original raw data); down-sampled at (**b**) 50 Hz; (**c**) 25 Hz; and (**d**) 10 Hz. Within the violin plot, the circular dot indicates the median. For all acceleration summary measures, the results are presented in gravitational units (g). We have standardized the scale for each of the acceleration summary measure in order to facilitate visual comparisons across the different sample rates.

Figure 2 presents the violin plots of the four acceleration summary measures (ENMONZ, MAD, AI, and ROCAM) for acceleration data sampled at 100, 50, 25 and 10 Hz, respectively. These plots serve to provide a visual overview of the differences in the distributions across the five PA categories within a specific sample rate (focusing on the comparisons within each of the four plots, i.e., Figure 2a–d) and also as a function of the sample rate in the accelerometry data (comparing the different acceleration summary measures across the different sample rates). By looking directly at the plots in Figure 2, we can visually appreciate that the range and distributions of values for all acceleration summary measures are different as a result of using accelerometry data sampled at different sample rates. This intuitively suggests that any thresholds and any statistical learning models developed with data collected when using a particular sample rate would likely not generalize well in a different dataset collected with a different sample rate. In other words, we need to have sample-rate-specific thresholds if we want to operate directly with data sampled at different sample rates.

In the following processing stages for the study, we used the acceleration data when sampled at 10 Hz. This is both because this is the sample rate that led to the overall highest correlation with the five categories (see Table 1) and because, practically, this is a more generalizable setting, given that we can always down-sample accelerometry data if they were collected at a higher sample rate (whereas we cannot recover information in the higher frequency ranges if we were to up-sample data).

**Figure 3:**
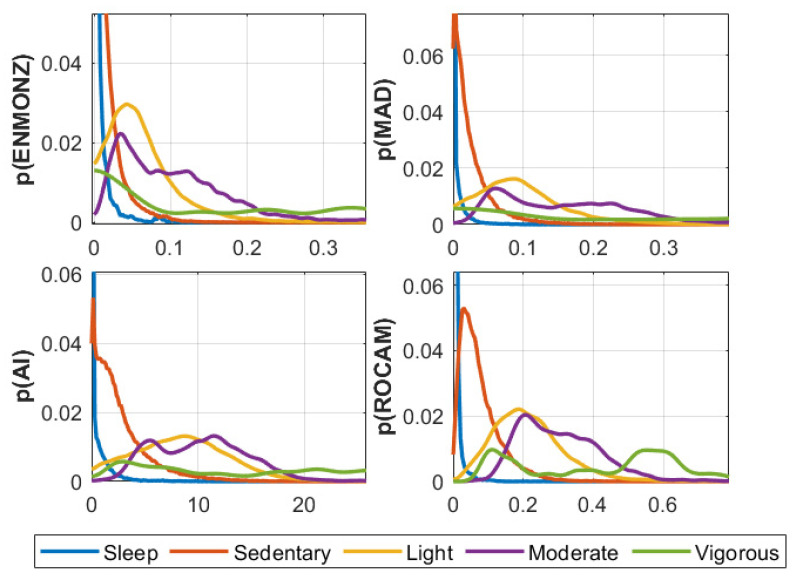
Probability distributions of the four acceleration summary measures (ENMONZ. MAD, AI, and ROCAM) for the five different categories in order to illustrate the distribution overlap. The plots are zoomed in to better appreciate possible thresholds we could be deriving (for an appreciation of the full range of the variable values, see Figure 2d). For brevity, we present only the results with the CAPTURE-24 data sampled at 10 Hz.

Figure 3 presents the densities for the four acceleration summary measures, where we can obtain a visual illustration of the extent of overlap and intuitively get a feel for possible thresholds we might be exploring to differentiate the five categories. There is considerable overlap across the five categories, particularly between sleep and sedentary, and between light, moderate, and vigorous PA. Visually it is not immediately clear if any of the four acceleration summary measures is superior, although ROCAM arguably has clearer points that we might use to define thresholds to differentiate the categories (e.g., vigorous PA stands out more clearly). We remark that, with such high correlation coefficients (in Table 1) and the illustrations in Figure 2 and Figure 3, we can be quite confident that we should be able to identify the PA categories correctly most of the time by using acceleration summary measure thresholds. Intuitively, these plots also serve to caution us about the differentiation of certain PA categories when using threshold-based methods, for example, where there is substantive overlap in the distributions (e.g., in light and moderate PA).

Table 2 summarizes the optimized thresholds for each of the four acceleration summary measures following the formal optimization process to maximize accuracy, along with their overall accuracy in differentiating the PA categories when using accelerometry data sampled at 10 Hz. For completeness, we also present the optimized thresholds when using data sampled at 25, 50, and 100 Hz in Supplementary Tables S1–S3, respectively, in the Supplementary Material. We remark that ROCAM has an edge over the competing acceleration summary measures, with a reported accuracy of 80.8%, thus verifying what we intuitively expected from the findings presented in Table 1 with the reported correlation coefficients.

A relevant question then is whether this difference in performance between ROCAM and the other acceleration summary measures is statistically significant. We used the two-sample KS test to assess whether the resulting estimates from the different acceleration summary measures are statistically significantly different. In all cases, we found that the results were statistically significantly different (*p* < 0.0001), and, combined with the results presented in Table 2, this indicates that ROCAM indeed offers a statistically significant improvement over its competitors in terms of accurately estimating the PA categories.

**Figure 4:**
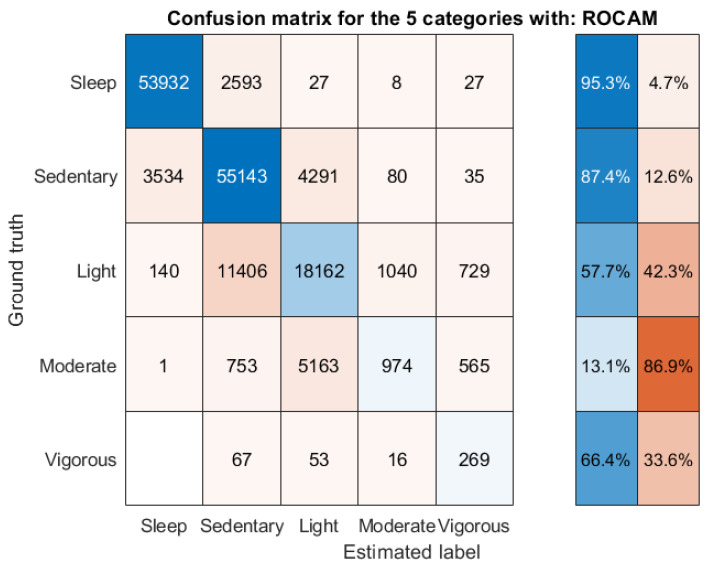
Minute-wise confusion matrix to estimate the five categories (sleep; sedentary, light, moderate, and vigorous PA) by using optimized thresholds for ROCAM with accelerometry data sampled at 10 Hz. On the right-hand side, we have the percentage of correctly vs. incorrectly matched labels for each of the five categories. Overall accuracy: 80.8%. The results refer to out-of-sample performance and were computed by using the leave-one-participant-out approach, wherein we collated all outputs in a single confusion matrix.

Figure 4 shows the confusion matrix for ROCAM that was used towards estimating the PA categories by using the optimized thresholds reported in Table 2. On the right-hand side, we also present the percentage of correctly vs. incorrectly matched labels for each of the five PA categories, as this serves to identify areas of potential improvement more easily (in combination with the actual entries off the main diagonal in the confusion matrix). For example, it is clear that sleep and sedentary activity are very accurately detected and there is space for improving the estimation of light and moderate activity; in particular, the ‘moderate PA’ is often mistakenly estimated as ‘light PA’. For comparison with the ROCAM findings presented in Figure 4, we also present the confusion matrices for ENMONZ, MAD, and AI in Supplementary Figures S1–S3. We note that there are some fairly substantive differences in the estimation of different PA categories amongst the different threshold-based methods for the acceleration summary measures. For example, ROCAM is particularly powerful in correctly identifying sleep, sedentary activity, and vigorous PA compared to ENMONZ, MAD, and AI, whereas all of these three competing methods are better at estimating light PA compared to ROCAM. These differences in the accuracy of estimating particular PA activities could implicitly suggest that some sort of combination or voting using all four acceleration summary measures and/or the estimated PA categories might lead to better results and, hence, motivates the following step.

**Figure 5:**
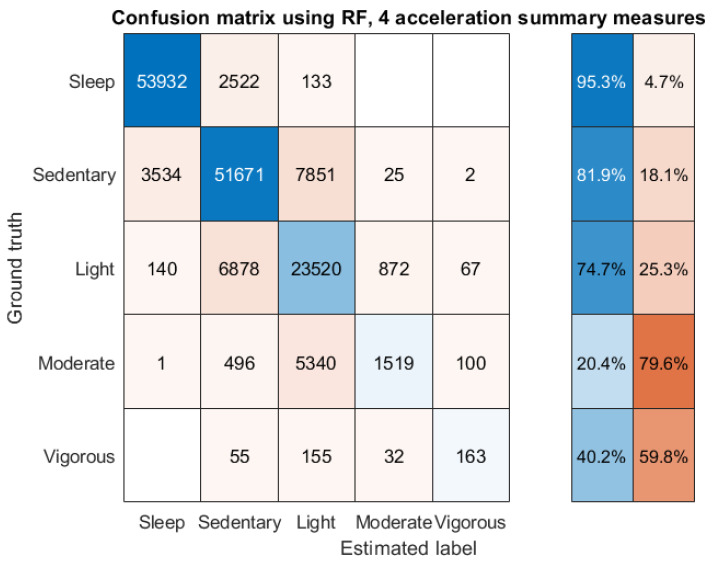
Minute-wise confusion matrix to estimate the five categories (sleep; sedentary, light, moderate, and vigorous PA) by using the optimized thresholds independently for each of the four acceleration summary measures and presenting their outputs (estimated categories) as inputs to an RF. On the right-hand side, we have the percentage of correctly vs. incorrectly matched labels for each of the five categories. Overall accuracy: 82.2%. The results refer to out-of-sample performance and were computed by using the leave-one-participant-out approach, wherein we collated all outputs in a single confusion matrix.

In an attempt to explore improving these findings, we used RF developing statistical learning models. Specifically, we (i) computed the estimated outputs for the five categories when applying the optimized thresholds for each of the four acceleration summary measures independently, and then we presented these outputs to an RF; and (ii, iii) combined the two steps presented above, presenting them jointly as inputs into an RF. Figure 5 presents the confusion matrix of the trained RF (model where the estimated PA outputs followed the application of the optimized thresholds): we observed that there is further improvement in overall accuracy (82.2%) that mainly comes from improving the estimates for the ‘light’ and ‘moderate’ PA categories. However, this comes at the cost of some reduction in the estimation accuracy of sedentary and vigorous PA. We remark that the results of the RF models built either with the use of the acceleration summary measures directly, or with the use of jointly the acceleration summary measures along with the PA estimates following the threshold application were similar to the RF model built using the PA estimates following the optimized threshold application. Therefore, we opted to present the approach that is computationally simplest.

## 4. Discussion

We investigated four acceleration summary measures (three widely used and the new ROCAM proposed here) across four different sample rates (100, 50, 25 and 10 Hz) to process three-dimensional acceleration data collected from the wrist in order to construct a 24-hour physical-activity-and-sleep-profile assessment. The study has made a number of key contributions. First, we have demonstrated that the widely used and practically default approach to summarize three-dimensional accelerometry data in actigraphy (ENMONZ) is likely not the best method to summarize the raw data, at least not towards the differential assessment of sleep and the standard PA categorization (sedentary, light, moderate, and vigorous PA), as reported in Table 1 and Table 2. Second, the new acceleration summary measure, ROCAM, appears to be a very competitive method, particularly towards the differentiation of sleep and PA, with the additional advantage that it is very robust with reduced sample rate (in fact, the association strength of ROCAM with the five categories is practically optimized with data sampled at 10 Hz). Third, we have shown that different sample rates for the three-dimensional accelerometry data can have a considerable effect in terms of the range in the acceleration summary measure values (see Figure 2) and, to a lesser extent, to the resulting statistical association with PA categories (Table 1); this has important implications for carefully considering how to use reported thresholds and built models across studies with different sample rates. Fourth, sampling the three-dimensional acceleration data at 10 Hz is fully sufficient for the purpose of differential assessment of sleep and PA, at least when it comes to using threshold approaches to estimate the five PA categories explored in this study. Fifth, by using ROCAM and appropriately optimized thresholds, we can obtain 80.8% accuracy in differentiating the PA categories; we can improve accuracy by considering all four acceleration summary measures and presenting them in an RF to obtain 82.2% accuracy in correctly matching the PA categories. We would especially like to highlight the very accurate detection of sleep and sedentary activity from using simple thresholds for ROCAM (see Figure 4), which has important implications in understanding ADL and their association with healthcare outcomes [4].

ENMONZ is an intuitively appealing acceleration summary measure which builds on the standard concept of Euclidean distance. This explains why it is practically the default option in actigraphy packages (such as GGIR) and its widespread use, including in the study that reported on the CAPTURE-24 data [31] and many other studies in the research literature [3,33,44]. However, the findings presented in this study challenge the status quo and the widespread use of ENMONZ. We have reported (see Table 1) that ENMONZ is the acceleration summary measure which has the lesser association strength with the five PA categories amongst the four acceleration summary measures investigated. Moreover, ENMONZ was the only one of the four acceleration summary measures that has a statistical association with the five categories that degrades considerably with data sampled at 10 Hz. This is not a problem in itself, necessarily, if an acceleration summary performs very well, say at 100 Hz, and degrades considerably when presented with low-resolution data; however, it is an indicator that, particularly with acceleration data sampled at low sample rates, one should be cautious when using ENMONZ. Particularly given that ENMONZ is not better associated with the PA categories at the higher sample rates, there is good evidence presented here to suggest that competing acceleration summary measures should be strongly considered for PA categorization and likely also for other actigraphy data-processing tasks. We reported that there is a small but clear performance improvement of 2% (which is also statistically significant) when using ROCAM over ENMONZ; this is noteworthy, given that this is a challenging five-class problem. The developers of MAD and AI had previously compared their findings to ENMONZ [21,22]; however, those comparisons were limited from using shorter-period laboratory-based data compared to the 24-hour data collected under free living conditions explored in this study. Therefore, the comparisons of the four acceleration summary measures (ENMONZ, MAD, AI, and ROCAM) reported here are novel in terms of the data-collection environment (non-controlled) and duration of collection, which, in principle, should better reflect the generalizability of the presented findings in other free-living longitudinal actigraphy studies.

Perhaps counter-intuitively, ROCAM appears to be more strongly associated with the five categories when using lower-resolution acceleration data (sampled at 10 Hz), as can be seen in Table 1. This likely reflects the instantaneous nature of the ROCAM algorithm, as it takes successive differences across each of the three axes, and, hence, it might be that operating at very high granularity (e.g., with the 100 Hz sampled data) might reflect changes in internal accelerometer noise. Therefore, this empirical finding likely suggests that ROCAM might be overly sensitive to very high-resolution data, and it is *advantageous* to down-sample the data at least for the purpose of estimating the five categories used herein. It will be interesting to see if this finding can be generalized for other settings where the actual labels in a problem are different (for example, trying to directly estimate different activities, as explored in Willetts et al. [33]).

Previous work has reported findings and provided acceleration summary measure thresholds or models to assess PA when using high-sampled acceleration data, e.g., 80 Hz [22] and 100 Hz [31,33], as this limits applicability in new datasets that do not use the same high sample rates since these thresholds and models are sample-rate dependent. Arguably, in most practical applications in community studies, we do not need a sample rate beyond 25 Hz for a general-purpose assessment of PA because typically people do not move their hands beyond a few times per second (at least in the sense of what we would be interested to assess in most research studies for PA). By Nyquist’s theorem [28], we should be aiming to sample at about twice the maximum frequency of interest in the data; therefore, a sample rate of 100 Hz would probably be considered excessive for research purposes focusing on the general population for longitudinal PA assessment. Given that there is usually a trade-off to be made between the use of the sample rate and the duration of the data collected (indicatively, Geneactiv can collect up to 7 days of data at 100 Hz and about 60 days of data at 10 Hz in a single charge; Axivity AX3 has a maximum logging period of 30 days at 12.5 Hz or 14 days at 100 Hz) and that many research studies often aim to provide longitudinal outcome assessments, a pragmatic practical decision would be to use 10 Hz to maximize data collection duration, whilst retaining meaningful signal variability on per-second basis. For these reasons, and to ensure that the provided algorithms developed herein are generalizable and deployable in studies planning to focus on longer-term monitoring, we wanted, in particular, to assess the use of three-dimensional data sampled at 10 Hz. The implication is that it is easy to down-sample accelerometry datasets which were collected by using a higher sample rate and directly use the methodology and the thresholds described in this study. On the contrary, a research study which used a sample rate of 10 Hz would not be able to benefit from guidelines and recommended thresholds and models developed if these require high-sample-rate data.

The differences in performance for accelerometry data sampled at different sample rates can be appreciated by comparing the results presented in Table 2 and the Supplementary Tables S1–S3, which present findings with data sampled at 25, 50, and 100 Hz, respectively. We note that, as expected from the correlation coefficient results reported in Table 1, the accuracy of obtaining the PA categories with appropriately optimized thresholds was best when using data sampled at 10 Hz, using ROCAM. A careful comparison of these tables also reveals important differences in the optimized thresholds when using acceleration data sampled at different sample rates for all the acceleration summary measures, a finding which is not unexpected when considering the probability distributions presented in Figure 2. This serves to highlight the importance of carefully considering the choice of sample rate on generalizing findings towards PA assessment. Overall, on the basis of the current evidence in this study, it appears that using three-dimensional accelerometry data sampled at 10 Hz is fully sufficient for the purposes of the differential assessment of PA categories explored here.

It is difficult to directly compare side-by-side the accuracy reported here with findings in the research literature, in part because some works use different PA categories, exclude sleep assessment (e.g., focusing on shorter time intervals rather than 24-hour profile assessment), or focus on lab-based recordings where very specific tasks are followed under controlled conditions to calculate METs. Walmsley et al. [3] were the first to use CAPTURE-24 data to assess different categories, similarly to this study, towards 24-hour profile assessment, and they reported a 87.7% accuracy. They used four categories (sleep, sedentary PA, light PA, and MVPA); that is, compared to this study, they had combined moderate and vigorous PAs into a single category (since there are relatively few samples in vigorous PA in the CAPTURE-24 dataset, and they wanted to use a balanced dataset for training the RF). They had retained the originally sampled 100 Hz data and had trained a complicated RF to estimate the four categories when presented with 50 advanced features extracted from the raw accelerometry data by applying a range of signal processing algorithms. This is a compelling approach: in practice, we can develop and apply different algorithms to characterize the accelerometry data and subsequently select a robust parsimonious feature subset (e.g., using feature selection or feature transformation methods [45,46]) which is presented to the statistical learner. Compared to Walmsley et al., we remark that the approach explored here is (a) generalizable across different sample rates and, in particular, can be run in emerging actigraphy datasets sampled at 10 Hz that we and others are currently collecting, thus practically enabling longer data collection on a single smartwatch charge compared to higher sample rates (we will be reporting on these findings in future follow-up work); (b) computationally much faster, since it does not involve the computation of many advanced features that are presented to a statistical learner (and potentially also involving additional steps in the statistical learning process such as feature selection or feature transformation); (c) differentiates between moderate and vigorous PA, as such a distinction is clinically important for certain applications and overall WHO recommendations regarding weekly exercise. We also stress that the use of threshold-based methods intrinsically avoids the well-known problem of supervised statistical learning setups when training on highly unbalanced datasets where the dominant class(es) will typically be the output of the classifier. Hence, in this study, we did not need to explicitly account for creating balanced subsets as in Walmsley et al. [3]. Instead, with threshold-based methods, we can control for potential clear dominance of classes by carefully setting the lower- and upper-boundaries in the constrained optimization algorithm where we compute the thresholds (in this study, these were set following visual inspection of the probability distributions; see Figure 3). In the results not shown in this study, we found that the resulting complicated trained RF of Walmsley et al. [3] does not generalize well in datasets sampled at different sample rates (i.e., when down-sampling the CAPTURE-24 data at 25 Hz or in new datasets we are currently in the process of collecting, sampled at 10 Hz). Intuitively, this could be explained by the differences we noted in the acceleration summary distributions reported when using acceleration data sampled at different sample rates (see Figure 2). Therefore, the requirement of having data sampled at 100 Hz to use their trained statistical leaning model is practically very restrictive for actigraphy studies aiming to collect longitudinal data.

From a practical perspective, the approach proposed here, with the use of acceleration summary thresholds, needs to be further improved in terms of correctly assessing light and moderate PA, which are conflated with sedentary and light PA, respectively (see Figure 4 and Figure 5). There is clear room for improvement, for example, by deploying some postprocessing approach, possibly invoking dynamic programming concepts or some other method that takes into account the actual time-state changes to more accurately follow the trajectory of the five categories for each participant. This is an area that we intend to build on further in future work. We note that other threshold-based PA categorization studies had similarly reported challenges in distinguishing between certain categories, for example, between sedentary and light PA and/or overestimating MVPA [25,44].

We note that the CAPTURE-24 is the largest known freely available accelerometry-based dataset with detailed minute-by-minute labels, where data have been collected under real-world conditions rather than in highly controlled lab settings. This makes it an ideal dataset upon which to develop and validate an algorithmic framework towards assessing 24-hour activity profiles, with the vision that the lessons learned can be translated to similar large-scale community studies. For example, the developers of CAPTURE-24 have used the learnings from their algorithmic framework in CAPTURE-24 and PA assessments to provide new insights into the UK BioBank study analyzing weekly actigraphy data from more than 85,000 participants [3]. The provided labels in the form of METs in CAPTURE-24 were estimated by reviewing photos and diaries rather than being directly measured, and therefore it is possible that the developed algorithms might need to be further refined with additional datasets where lab-based METs measurements are available. Nevertheless, for the purposes of obtaining an overall PA categorization (including sleep) and 24-hour profile assessment under non-controlled lab conditions, the CAPTURE-24 dataset is a particularly useful resource.

The study has a number of limitations which we acknowledge. Although CAPTURE-24 is the largest publicly available actigraphy database in free living conditions *with detailed labels*, findings ideally need to be explored and validated on a larger cohort or additional external datasets. In particular, it would be useful to have cohorts with different pathologies, since algorithms developed when processing actigraphy data in healthy controls do not necessarily generalize well in regard to people with certain pathologies, e.g., sleep-related pathologies [18]. We focused on four acceleration summary measures (ENMONZ, MAD, AI, and ROCAM), motivated by the use of the first three computational approaches which have been widely used in similar reports, and proposing the new acceleration summary measure ROCAM. There are other acceleration summary measures which have been proposed in the research literature which have not been explored here due to space and practicality constraints and the fact they had not shown any tangible advantages over the acceleration summary measures reported here (see [20], for example). Furthermore, there are some devices (and also legacy datasets and legacy algorithms) operating on ‘activity counts’, often focusing on mono-axial accelerometers (i.e., single-axis acceleration), where a popular acceleration summary measure is zero crossing (ZC). There is existing work to align recent developments in three-dimensional acceleration processing and count-based or ZC algorithms to enable backwards compatibility [47], and these approaches and acceleration summary measures were not investigated herein because, arguably, they offer a more crude measure of activity compared to sensors, which provide raw three-dimensional acceleration signals. The methods proposed herein were validated on participants wearing the accelerometer on their dominant hand; cutoffs to assess PA levels are likely dependent on device placement (dominant or non-dominant hand; see [15,48]), and, hence, future work should carefully consider how well the findings can be generalized. Moreover, the findings presented in this study are for *wrist-worn* acceleration data; although the methodology presented should, in principle, be applicable to other body placements, specific detailed thresholds and findings reported herein would be different for acceleration data recorded elsewhere (e.g., on the hip), as previous work has shown [15,24,49]. Finally, we did not consider age-, gender-, and motor-competence-specific analyses due to the relatively limited sample size to perform these detailed stratifications: there is some work that suggests that these affect accelerometer outputs, calling for more personalized threshold-based methods [50,51].

## 5. Conclusions

Collectively, this study contributed new insights into the analysis of wrist-worn actigraphy data in three areas. Firstly, it introduced a new robust acceleration summary measure (ROCAM) which was shown to be very competitive against three established and widely used acceleration summary measures (ENMONZ, MAD, and AI) in terms of summarizing the data in 1-minute epochs that can be subsequently mapped onto sleep and PA activities. For the first time, these thorough acceleration summary measure comparisons were obtained by processing a large dataset collected under free-living conditions (CAPTURE-24) where we have access to 24-hour minute-wise labeled actigraphy data. Secondly, it provided solid evidence that acceleration data with a 10 Hz resolution is fully sufficient for sleep and differential PA assessment, thus paving the way for the adoption of the 10 Hz sample rate in actigraphy studies which aim to focus on longitudinal sleep and PA analysis, thus minimizing the need for regular device recharge. Finally, it demonstrated a straightforward, reasonably accurate, and clearly interpretable threshold-based methodology to infer sleep and the four standard PA categories (sedentary, light, moderate, and vigorous) which are widely used when processing actigraphy data. In particular, we have shown that the estimation of sleep and sedentary activity are very accurately estimated, both of which have important healthcare implications.

## Figures and Tables

**Table 1:** Spearman correlation coefficients of the four acceleration summary measures with the five activity categories (sleep; sedentary, light, moderate, and vigorous PA) when processing three-dimensional acceleration data at different sample rates.

	(Hz)	ENMONZ	MAD	AI	ROCAM
**Data sampled at (Hz)**	**100**	0.760	0.837	0.845	**0.861**
	**50**	0.761	0.844	0.848	**0.872**
	**25**	0.758	0.845	0.848	**0.883**
	**10**	0.737	0.843	0.844	**0.884**

PA stands for Physical Activity, ENMONZ for Euclidean Norm Minus One Non-Zero, MAD for mean amplitude deviation, AI for Activity Index, and ROCAM for Rate Of Change Acceleration Movement (the latter being a new algorithm proposed in this study). For the algorithmic definitions of these four accelerometer summary measures, see Equations (1)–(4) in the text. For the MATLAB implementation computing those, see https://github.com/ThanasisTsanas/ActigraphyToolbox (last accessed on 30 June 2022).

**Table 2:** Thresholds to differentiate the different PA categories for the four acceleration summary measures and resulting accuracy with accelerometry data sampled at 10 Hz.

	ENMONZ	MAD	AI	ROCAM
**Sleep**	Estimated by using a separate sleep detection algorithm and additional entries that are below the lowest threshold of sedentary activity for each of the acceleration summary measures			
**Sedentary PA**	0 < x ≤ 0.032	0.001 < x ≤ 0.059	0.010 < x ≤ 5.308	0.06 < x ≤ 0.175
**Light PA**	0.032 < x ≤ 0.173	0.059 < x ≤ 0.242	5.308 < x ≤ 17.010	0.175 < x ≤ 0.400
**Moderate PA**	0.173 < x ≤ 0.382	0.242 < x ≤ 0.38	17.010 < x ≤2 3.628	0.400 < x ≤ 0.483
**Vigorous PA**	x > 0.382	x > 0.38	x > 23.628	x > 0.483
**Accuracy (%)**	78.8	79.6	78.4	**80.8**

PA stands for Physical Activity. For determining sleep, we used a slightly modified algorithm that we had previously proposed [18] (see text for details). Using the sleep detection algorithm and the PA thresholds to estimate the five categories leads to the computed accuracies reported herein. For all acceleration summary measures, the results are presented in gravitational units (g).

## Data Availability

The dataset used in this study is publicly available from https://ora.ox.ac.uk/objects/uuid:99d7c092-d865-4a19-b096-cc16440cd001. (https://doi.org/10.5287/bodleian:NGx0JOMP5).

## Supplementary Materials

The following supporting information can be downloaded at: https://www.mdpi.com/article/10.3390/s22166152/s1, Table S1: Thresholds to differentiate the different PA categories for the four acceleration summary measures and resulting accuracy with accelerometry data sampled at 25 Hz. Table S2: Thresholds to differentiate the different PA categories for the four acceleration summary measures and resulting accuracy with accelerometry data sampled at 50 Hz. Table S3: Thresholds to differentiate the different PA categories for the four acceleration summary measures and resulting accuracy with accelerometry data sampled at 100 Hz. Figure S1: Minute-wise confusion matrix to estimate the five categories (sleep, sedentary, light, moderate, vigorous) using optimized thresholds for ENMONZ with accelerometry data sampled at 10 Hz. On the right hand-side we have the percentage of correctly vs incorrectly matched labels for each of the five categories. Overall accuracy: 78.8%. The results refer to out-of-sample performance and were computed using leave-one-participant-out where we collated all outputs in a single confusion matrix. Figure S2: Minute-wise confusion matrix to estimate the five categories (sleep, sedentary, light, moderate, vigorous) using optimized thresholds for MAD with accelerometry data sampled at 10 Hz. On the right hand-side we have the percentage of correctly vs incorrectly matched labels for each of the five categories. Overall accuracy: 79.6%. The results refer to out-of-sample performance and were computed using leave-one-participant-out where we collated all outputs in a single confusion matrix. Figure S3: Minute-wise confusion matrix to estimate the five categories (sleep, sedentary, light, moderate, vigorous) using optimized thresholds for AI with accelerometry data sampled at 10 Hz. On the right hand-side we have the percentage of correctly vs incorrectly matched labels for each of the five categories. Overall accuracy: 78.4%. The results refer to out-of-sample performance and were computed using leave-one-participant-out where we collated all outputs in a single confusion matrix.

## Source Code Availability

https://github.com/ThanasisTsanas/ActigraphyToolbox.
