# Supplementary material for: Investigating Wrist-Based Acceleration Summary Measures across Different Sample Rates towards 24-Hour Physical Activity and Sleep Profile Assessment
Source: Sensors (Basel). 2022 Aug 17;22(16):6152. doi: 10.3390/s22166152 (PMC9413015; doi:10.3390/s22166152)
Supplement: Supplementary file 1 [file sensors-22-06152-s001.zip › sensors-1821383-supplementary.pdf]

Electronic Supplementary Material

Investigating wrist-based accelerometry summary measures across different sample rates towards 24-hour physical activity and sleep profile assessment

Optimized thresholds at different sample rates

In Table 2 of the manuscript we presented the optimized thresholds when using accelerometry data sampled at 10 Hz. Supplementary Tables S1, S2, and S3 provide the optimized thresholds when using accelerometry data sampled at 25, 50 and 100 Hz, respectively. We observe that the performance of ROCAM *improves* with the use of accelerometry data sampled at smaller sample rates, which was expected following the reported correlation coefficients in Table 1 in the main manuscript. ENMONZ has the greatest stability, in the sense that the overall performance changes very slightly as a function of the sample rate. The other three acceleration summary measures exhibit a more variable overall performance as a function of the sample rate.

Collectively for the findings reported in Tables S1, S2 and S3, we postulate that ROCAM, because of its design as a rate of change accelerometry-based measure, may be more severely affected by internal (thermal) noise from the accelerometer as high sample rates: internal noise affects proportionally more strongly these rate of change differences when using a high resolution. We elaborate further about this finding in the main manuscript. Further work will need to more rigorously assess this hypothesis, likely with multiple accelerometer devices recorded concurrently in a standard lab setting where we can control accelerometer movement.

Table S1. Thresholds to differentiate the different PA categories for the four acceleration summary measures and resulting accuracy with accelerometry data sampled at 25 Hz.

	**ENMONZ**	**MAD**	**AI**	**ROCAM**
**Sleep**	Estimated using a separate sleep detection algorithm and additional entries that are below the lowest threshold of sedentary activity for each of the acceleration summary measures			
**Sedentary PA**	0<x≤0.053	0.002<x≤0.071	0.168<x≤6.42	0.009<x≤0.124
**Light PA**	0.053<x≤0.181	0.071<x≤0.274	6.42<x≤15.82	0.124<x≤0.326
**Moderate PA**	0.181<x≤0.308	0.274<x≤0.395	15.82<x≤21.56	0.326<x≤0.373
**Vigorous PA**	x>0.308	x>0.395	x>21.56	x>0.373
**Accuracy (%)**	78.2	80.2	78.8	80.2

PA stands for Physical Activity. For determining sleep we used a slightly modified algorithm that we had previously proposed [1] (see text in the main manuscript for details). Using the sleep detection algorithm and the PA thresholds to estimate the five categories leads to the computed accuracies reported herein. For all acceleration summary measures the results are presented in gravitational units (g).

Table S2. Thresholds to differentiate the different PA categories for the four acceleration summary measures and resulting accuracy with accelerometry data sampled at 50 Hz.

	ENMONZ	MAD	AI	ROCAM
**Sleep**	Estimated using a separate sleep detection algorithm and additional entries that are below the lowest threshold of sedentary activity for each of the acceleration summary measures			
**Sedentary PA**	0 <x≤0.046	0.<x≤0.080	0.263<x≤6.810	0.04<x≤0.091
**Light PA**	0.046<x≤0.185	0.080<x≤0.281	6.810<x≤18.640	0.091<x≤0.250
**Moderate PA**	0.185<x≤0.320	0.281<x≤0.38	18.640<x≤24.127	0.250<x≤0.274
**Vigorous PA**	x>0.320	x>0.38	x>24.127	x>0.274
**Accuracy (%)**	78.5	79.0	79.2	77.5

PA stands for Physical Activity. For determining sleep we used a slightly modified algorithm that we had previously proposed [1] (see text in the main manuscript for details). Using the sleep detection algorithm and the PA thresholds to estimate the five categories leads to the computed accuracies reported herein. For all acceleration summary measures the results are presented in gravitational units (g).

Table S3. Thresholds to differentiate the different PA categories for the four acceleration summary measures and resulting accuracy with accelerometry data sampled at 100 Hz.

	ENMONZ	MAD	AI	ROCAM
**Sleep**	Estimated using a separate sleep detection algorithm and additional entries that are below the lowest threshold of sedentary activity for each of the acceleration summary measures			
**Sedentary PA**	0<x≤0.044	0<x≤0.078	0.134<x≤7.412	0.008<x≤0.053
**Light PA**	0.044<x≤0.178	0.078<x≤0.241	7.412<x≤31.130	0.053<x≤0.150
**Moderate PA**	0.178<x≤0.356	0.241<x≤0.425	31.130<x≤41.800	0.150<x≤0.369
**Vigorous PA**	x>0.356	x>0.425	x>41.800	x>0.369
**Accuracy (%)**	78.3	78.9	78.7	77.5

PA stands for Physical Activity. For determining sleep we used a slightly modified algorithm that we had previously proposed [1] (see text in the main manuscript for details). Using the sleep detection algorithm and the PA thresholds to estimate the five categories leads to the computed accuracies reported herein. For all acceleration summary measures the results are presented in gravitational units (g).

Comparing confusion matrices for the acceleration summary measures

In Figure 4 of the manuscript we presented the confusion matrix when using ROCAM with the accelerometry data sampled at 10 Hz. The overall accuracy for the different acceleration summary measures was presented at the bottom row of Table 2, where we have shown that ROCAM outperforms the competing acceleration summary measures in terms of overall accuracy. Supplementary Figures S1, S2, and S3 provide the corresponding confusion matrices for ENMONZ, MAD, and AI, respectively, to enable readers draw meaningful comparisons for the different acceleration summary measures.

We remark that there are some differences noteworthy for the estimation of different PA categories amongst the threshold-based outputs for the different acceleration summary measures. Overall, ROCAM has a clear edge in terms of estimating sleep, sedentary PA and vigorous PA (see Figure 4 in the manuscript compared to the confusion matrices reported in Figures S1-S3).

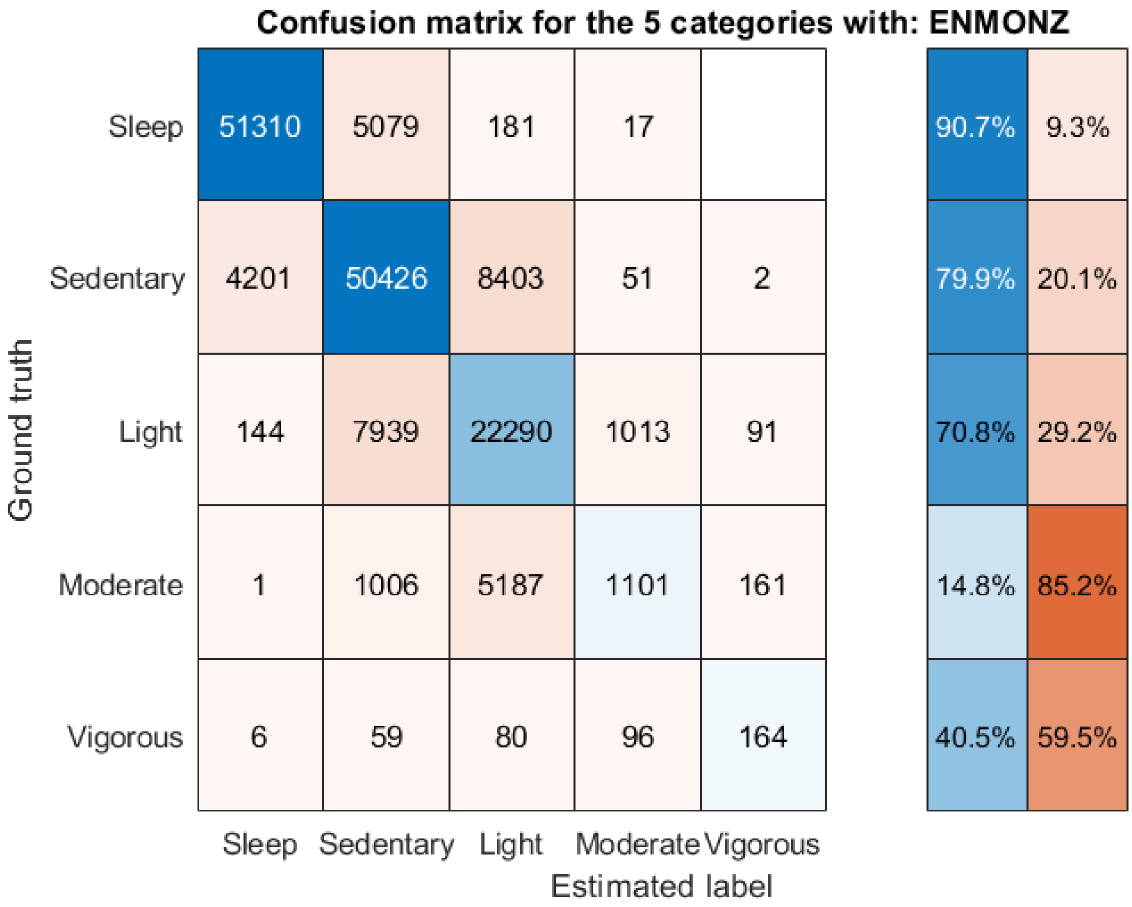

Figure S1. Minute-wise confusion matrix to estimate the five categories (sleep, sedentary, light, moderate, vigorous) using optimized thresholds for ENMONZ with accelerometry data sampled at 10 Hz. On the right hand-side we have the percentage of correctly vs incorrectly matched labels for each of the five categories. Overall accuracy: 78.8%. The results refer to out-of-sample performance and were computed using leave-one-participant-out where we collated all outputs in a single confusion matrix.

For some of the PA categories we note that e.g. using the MAD acceleration summary measure leads to better estimation accuracy of light PA and moderate PA compared to when we used ROCAM. Particularly for the estimation of the light PA remark that all the competing acceleration summary measures were somewhat better than ROCAM, which many times mis-assessed that PA category for sedentary PA. All approaches severely underperformed when estimating moderate PA. As noted in the main manuscript, it is precisely these types of differences in the estimation accuracy of the different PA categories for the use of the different acceleration summary measures that inspired the use of an approach to combine them (as reported in Figure 5).

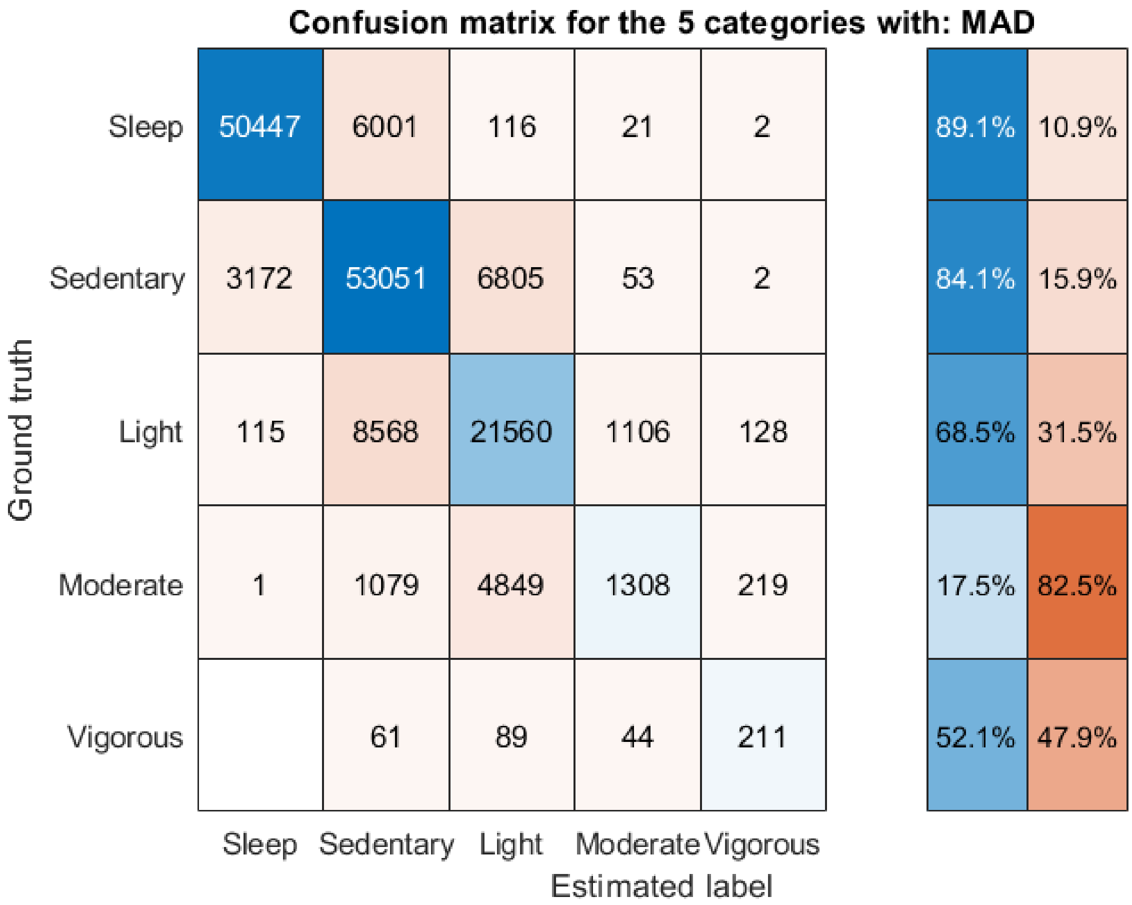

Figure S2. Minute-wise confusion matrix to estimate the five categories (sleep, sedentary, light, moderate, vigorous) using optimized thresholds for MAD with accelerometry data sampled at 10 Hz. On the right hand-side we have the percentage of correctly vs incorrectly matched labels for each of the five categories. Overall accuracy: 79.6%. The results refer to out-of-sample performance and were computed using leave-one-participant-out where we collated all outputs in a single confusion matrix.

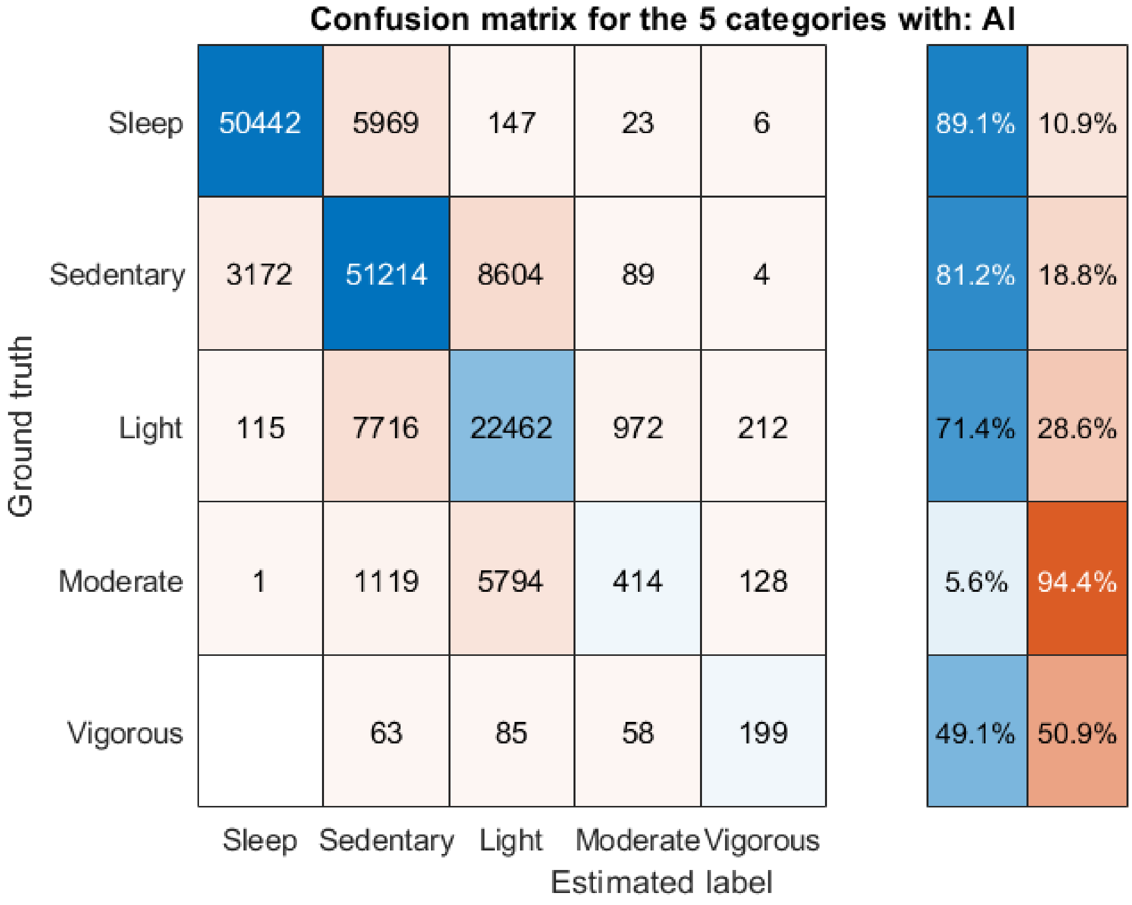

Figure S3. Minute-wise confusion matrix to estimate the five categories (sleep, sedentary, light, moderate, vigorous) using optimized thresholds for AI with accelerometry data sampled at 10 Hz. On the right hand-side we have the percentage of correctly vs incorrectly matched labels for each of the five categories. Overall accuracy: 78.4%. The results refer to out-of-sample performance and were computed using leave-one-participant-out where we collated all outputs in a single confusion matrix.

References

1. Tsanas, A.; Woodward, E.; Ehlers, A. Objective Characterization of Activity, Sleep, and Circadian Rhythm Patterns Using a Wrist-Worn Actigraphy Sensor: Insights into Post-Traumatic Stress Disorder. *JMIR mHealth uHealth* **2020**, *8*, e14306, doi:10.2196/14306.
